# CYP71D8 and CYP82A2 catalyze the last committed step in biosynthesis of glyceollin isomers in soybean

**DOI:** 10.5511/plantbiotechnology.24.1113a

**Published:** 2025-03-25

**Authors:** Tomoyoshi Akashi, Kai Uchida, Toshio Aoki

**Affiliations:** 1 Department of Applied Biological Sciences, Nihon University, Fujisawa, Kanagawa 252-0880, Japan

**Keywords:** cytochrome P450, glyceollin, *Glycine max*, phytoalexin

## Abstract

Glyceollins, which are prenylated pterocarpan phytoalexins found in soybean, play important roles in plant–microbe interactions. During biosynthesis, the formation of the cyclic ether ring from the C-5 prenyl side chain provides structural diversity to the glyceollin isomers. This reaction has been attributed to cytochrome P450 (P450); however, it is unclear whether a single enzyme or multiple enzymes are involved in glyceollin isomer formation. In this study, we searched a co-expressed gene network database for soybean. Known genes involved in glyceollin biosynthesis were used as queries, and eight P450s (CYP71D8, CYP81E24, CYP82A2, CYP82A3, CYP82A4, CYP93A2, CYP93A3, and CYP736A33) were selected as candidates. In vitro enzyme assays using recombinant yeast microsomes expressing P450s revealed that CYP71D8 produced glyceollin I from 4-dimethylallylglycinol, and CYP82A2 yielded glyceollin III from 2-dimethylallylglycinol. Real-time PCR analysis showed that transcripts of *CYP71D8* and *CYP82A2* were transiently induced in soybean cells upon elicitation, prior to the accumulation of glyceollins. Thus, CYP71D8 and CYP82A2 were identified as glyceollin I and glyceollin III synthases, respectively, indicating that distinct P450s catalyze the final steps in the biosynthesis of glyceollin isomers.

## Introduction

**Figure 1:**
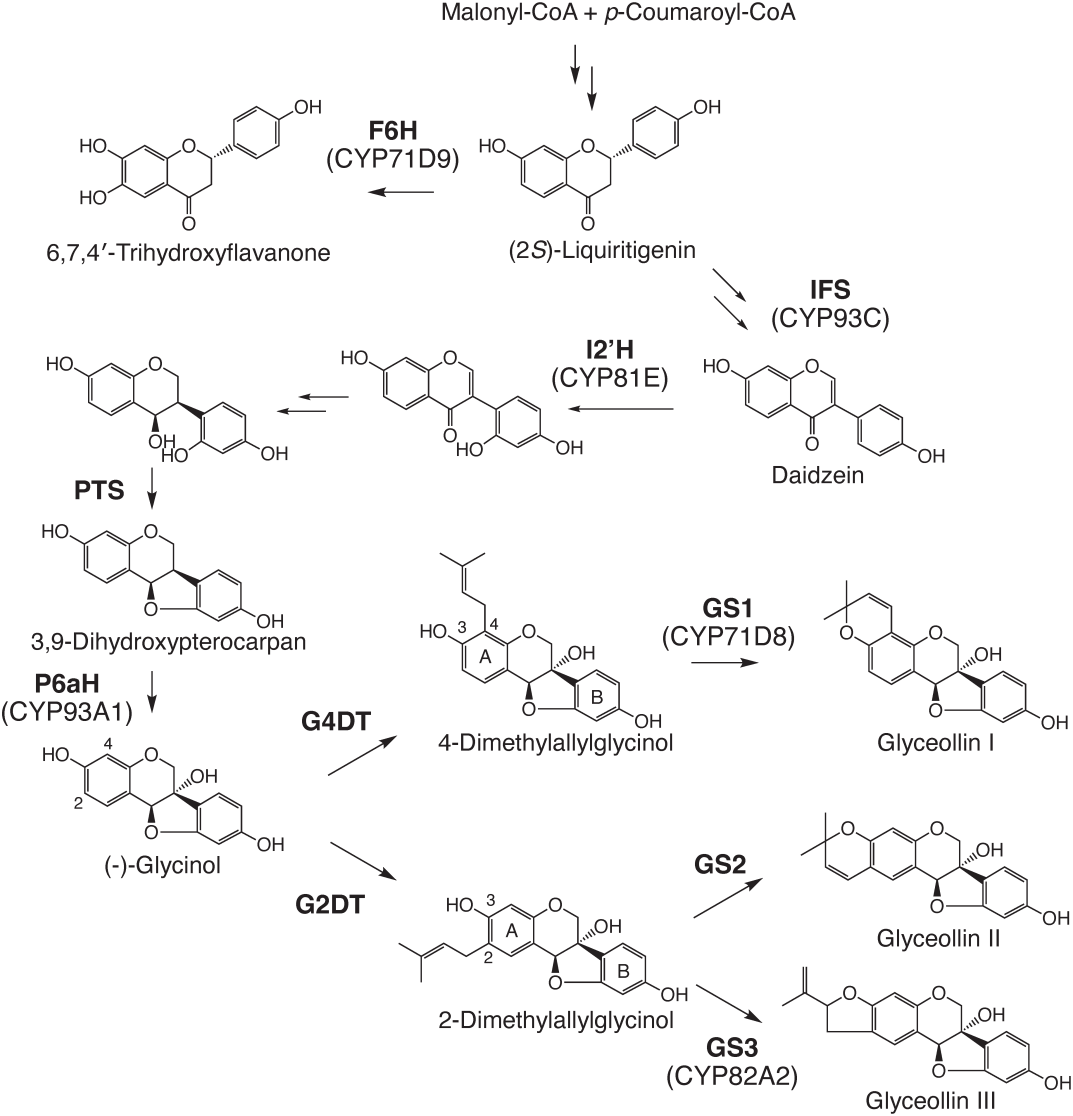
Figure 1. Biosynthesis of glyceollins. Abbreviations used are: F6H, flavonoid 6-hydroxylase; G2DT, (−)-glycinol 2-dimethylallyltransferase; G4DT, (−)-glycinol 4-dimethylallyltransferase; GS1, glyceollin I synthase; GS2, glyceollin II synthase; GS3, glyceollin III synthase; I2′H, isoflavone 2′-hydroxylase; IFS, isoflavonoid synthase; P6aH, pterocarpan 6a-hydroxylase; PTS, pterocarpan synthase.

Phytoalexins are antimicrobial secondary metabolites that are synthesized de novo by plants in response to biotic and abiotic stresses. Soybean (*Glycine max*) accumulate isoflavonoid phytoalexins known as glyceollins. Glyceollins possess a pterocarpan skeleton with five- or six-membered cyclic ether rings originating from the C5 prenyl moiety (Tahara and Ibrahim 1995). Prenylation of isoflavonoids leads to structural complexity and is believed to improve lipid solubility, membrane permeability, and enhance biological activities (Botta et al. 2005). Glyceollin I, II, and III are the major isomers, and a pterocarpan (−)-glycinol is a common precursor in their biosynthesis (Figure 1). To date, all enzymes involved in the biosynthesis of (−)-glycinol from (2*S*)-liquiritigenin have been characterized (Akashi et al. 2009; Ayabe et al. 2010; Dixon 1999; Uchida et al. 2017). The subsequent final two committed enzymatic steps, prenylation and cyclization, provide the structural diversity of glyceollin isomers. (−)-Glycinol 4-dimethylallyltransferase (G4DT) and (−)-glycinol 2-dimethylallyltransferase (G2DT) yielding precursors of glyceollin I, II, and III have been identified (Akashi et al. 2009; Sukumaran et al. 2018; Yoneyama et al. 2016). G4DT and G2DT are paralogs that arose through whole-genome duplications in soybean and show strict regiospecificity in prenylation reactions (Yoneyama et al. 2016). The formation of ether rings from the dimethylallyl moiety with the C-3 hydroxyl of the A-ring has been attributed to cytochrome P450 (P450) (Welle and Grisebach 1988). However, it is unclear whether single or multiple enzymes are involved in glyceollin isomer formation.

In this study, we characterized two P450s that catalyze the formation of glyceollin I from 4-dimethylallylglycinol (tentatively named glyceollin I synthase, GS1) and glyceollin III from 2-dimethylallylglycinol (glyceollin III synthase, GS3) using a co-expressed gene network data and subsequent enzyme assays with a recombinant yeast expression system.

## Materials and methods

### Chemicals

Glyceollins and 2- and 4-dimethylallylglycinols were sourced from our laboratory stocks (Akashi et al. 2009; Yoneyama et al. 2016).

### Selection of candidate P450 cyclases

Candidate genes were selected using a “find new members of a pathway” method in a soybean co-expression gene network database (SoyNet, https://www.inetbio.org/soynet/) (Kim et al. 2017) using CYP93C1 (isoflavonoid synthase, IFS), CYP81E11 (isoflavone 2′-hydroxylase, I2′H), and pterocarpan synthase (PTS) as queries. Among the listed co-expressed genes, P450s with unknown functions were selected.

### cDNA cloning

The open reading frames of the candidate P450s were amplified using PrimeSTAR MAX DNA polymerase (Takara Bio, Shiga, Japan), specific primers (Supplementary Table S1), and a cDNA template from black soybean (Uchida et al. 2015). For the TA cloning, overhanging dA at the 3′-ends were performed using ExTaq polymerase (Takara Bio). The products were then introduced into the pYES2.1 TOPO vector (Invitrogen, Carlsbad, CA, USA; CYP71D8, CYP71D145, CYP81E24, CYP82A2, CYP82A3, CYP82A4, CYP93A2, CYP93A3, and CYP736A33). CYP71D106 and CYP82A24 were synthesized using artificial gene synthesis (Eurofins, Tokyo, Japan) and introduced into the *BamH* I site of pYES2 vector using In-Fusion HD cloning kit (Takara Bio).

### Enzyme assays

For the expression of P450s in recombinant yeast cells, co-expression of cytochrome P450 reductase (CPR) is important for enhancing P450 activity (Seki et al. 2008). Yeast expression vectors were introduced into the protease-deficient *Saccharomyces cerevisiae* strain BJ2168, which harbored *Lotus japonicus* CPR cDNA (pESC-LEU) (Seki et al. 2008). The induction of recombinant proteins was performed as previously described (Ayabe et al. 2002).

The buffer used was 0.1 M potassium phosphate buffer (pH 7.5) containing 10% (w/v) sucrose and 14 mM 2-mercaptoethanol. The recombinant yeast cells suspended in the buffer were disrupted by shaking with glass beads. Crude extracts (10,000 g supernatant) were prepared by centrifugation (4°C, 10 min). The resultant crude extracts were ultracentrifuged at 160,000 g for 90 min. The final microsomal precipitates were homogeneously suspended in the same buffer as above. Enzyme assays were performed using crude extracts or recombinant yeast microsomes (188 µl), 10 µg of substrate (in 4 µl of 2-methoxyethanol), and 1 mM NADPH (in 8 µl of the buffer) in the total volume of 200 µl at 30°C for 20 min to 24 h. The ethyl acetate extract of the reaction mixture was analyzed by HPLC using an XBridge C18 column (4.6×150 mm, Waters, Milford, USA) or TSK-Gel ODS-80TM column (4.6×150 mm; Tosoh, Tokyo, Japan) at 40°C, with a flow rate of 1.0 ml min^−1^ and a linear gradient elution from 40 to 100% methanol over 20 min. The eluates were monitored at 280 nm. For kinetic studies, varying concentrations (1 to 30 µM) of substrate and a fixed concentration of NADPH (2 mM) were incubated with recombinant yeast microsomes (100 µg) expressing CYP71D8 and CYP82A2 in a total volume of 200 µl at 30°C for 20 min. Kinetic parameters were calculated using the Michaelis–Menten equation with SigmaPlot 14 software (Hulinks, Tokyo, Japan).

### Expression analysis

Expression analysis using real-time PCR was performed according to a previous study (Uchida et al. 2017), with primer sets listed in Supplementary Table S1.

### Phylogenetic tree analysis

The amino acid sequences of P450s were aligned using ClustalW, and a phylogenetic tree was generated by MEGA X using the neighbor-joining method with default settings (Kumar et al. 2018).

### Gene IDs

The IDs in the soybean genome (*G. max* Wm82.a2.v1) of the genes used in this study are as follows: CYP71D8, Glyma.11G062600; CYP71D9, Glyma.18G080400; CYP71D106, Glyma.01G179700; CYP71D145, Glyma.11G062500; CYP81E11, Glyma.15G156100; CYP81E24, Glyma.11G051800; CYP82A2, Glyma.13G285300; CYP82A3, Glyma.13G068800; CYP82A4, Glyma.01G135200; CYP82A24, Glyma.15G203500; CYP93A1, Glyma.03G143700; CYP93A2, Glyma.19G144700; CYP93A3, Glyma.03G142100; CYP93C1, Glyma.13G173500; CYP736A33, Glyma.07G089800; PTS, Glyma.03G147700.

## Results

### Selection of candidate P450s and in vitro enzyme assays

To identify candidate P450s, the co-expression gene network database of soybean (SoyNet) was searched. Three genes involved in glyceollin biosynthesis [CYP93C1 (IFS), CYP81E11 (I2′H), and PTS] were used as queries. Uncharacterized P450 genes co-expressed in all three queries (CYP71D8 and CYP82A4), two queries (CYP82A2), and one query (CYP81E24, CYP82A3, CYP93A2, CYP93A3, and CYP736A33) were selected as candidates.

**Figure 2:**
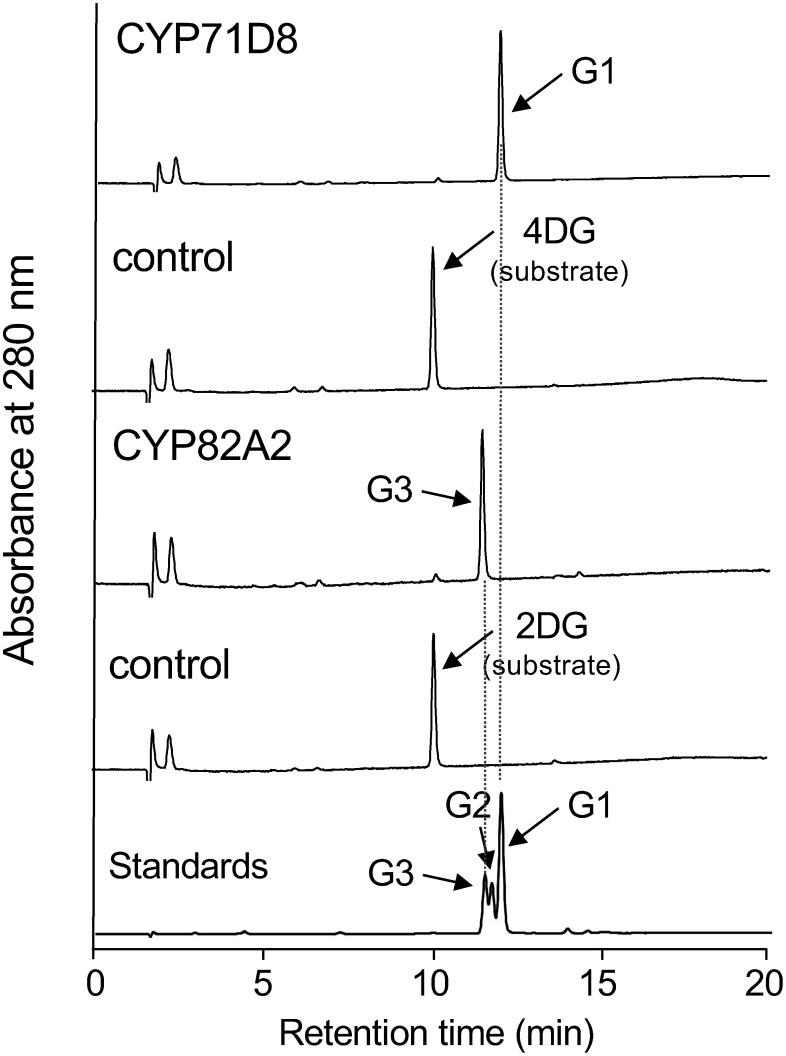
Figure 2. HPLC chromatogram of the enzymatic reaction mixture of CYP71D8 and CYP82A2. The yeast microsomes (160,000 g pellet) expressing P450s were reacted with a substrate (2- or 4-dimethylallylglycinol, 10 µg) in the presence of 1 mM NADPH at 30°C for 20 min. The ethyl acetate extract of the reaction mixture was analyzed by HPLC. For the control, the microsomal fraction of yeast cells transformed with pYES2 without a cDNA insert was used with the same substrates. The ordinate scales of the HPLC charts are equal. XBridge C18 column was used for the analysis. The eluates were monitored at 280 nm. Retention times are as follows: 2DG and 4DG (10.1 min), G1 (12.0 min), G2 (11.7 min), G3 (11.5 min). Abbreviations used are: 2DG, 2-dimethylallylglycinol; 4DG, 4-dimethylallylglycinol; G1, glyceollin I; G2, glyceollin II; G3, glyceollin III.

Initially, candidate P450s were expressed in yeast cells, and in vitro enzyme assays were performed using the crude extracts of yeast cells (10,000 g supernatant) and substrates (2- or 4-dimethylallylglycinols) in the presence of NADPH. The reaction mixtures of CYP71D8 with 4-dimethylallylglycinol and CYP82A2 with 2-dimethylallylglycinol produced single products, glyceollin I and glyceollin III, respectively (Supplementary Figure S1). Enzymatic assays were also performed using recombinant yeast microsomes with substrates (Figure 2, Supplementary Figure S2). The structures of both products recovered from the large-scale enzyme assays were further confirmed by nuclear magnetic resonance spectroscopy (Supplementary Data S1) (Akashi et al. 2009). Therefore, CYP71D8 and CYP82A2 were designated as GS1 and GS3, respectively. The other candidate P450s did not exhibit enzymatic activity toward 2- and 4-dimethylallylglycinols (Supplementary Figure S1).

To further characterize enzyme properties, the steady-state kinetic parameters of CYP71D8 and CYP82A2 were determined. The apparent *K*_m_ and *V*_max_ values of CYP71D8 for 4-dimethylallylglycinol were 1.3±0.2 µM and 510±103 pmol min^−1^ mg^−1^, respectively (Supplementary Figure S3). CYP82A2 exhibited a higher *K*_m_ (9.5±1.0 µM) and a similar *V*_max_ (420±35 pmol min^−1^ mg^−1^) compared to CYP71D8. The *K*_m_ value of CYP71D8 toward 4-dimethylallylglycinol (2 µM) was nearly the same as that of the microsomes from elicited soybean cells (Welle and Grisebach 1988).

### Search of paralogous genes

**Figure 3:**
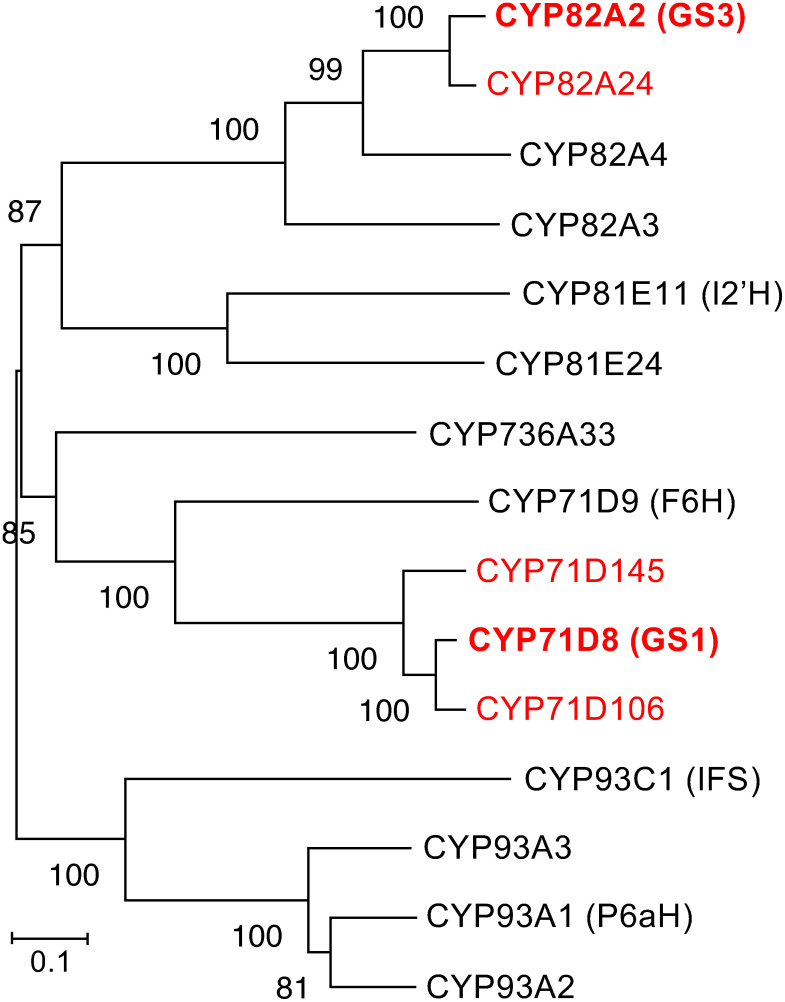
Figure 3. Phylogenetic relationships among P450s involved in soybean phytoalexin biosynthesis. Abbreviations used are: F6H, flavonoid 6-hydroxylase; GS1, glyceollin I synthase; GS3, glyceollin III synthase; I2′H, isoflavone 2′-hydroxylase; IFS, isoflavonoid synthase; P6aH, pterocarpan 6a-hydroxylase. Red letters indicate GS1, GS2, and their paralogous P450s analyzed in this study.

A protein BLAST was conducted using CYP71D8 and CYP82A2 as queries to identify additional glyceollin synthases in the soybean genome. The obtained candidates were subjected to phylogenetic tree analysis (Figure 3). CYP71D106 and CYP71D145 formed a monophyletic clade with CYP71D8, with amino acid identities greater than 84% (Supplementary Figure S4). CYP82A24 exhibited 92% amino acid identity with CYP82A2 and was closely related to CYP82A2 in the CYP82A subfamily.

CYP71D106, CYP71D145, and CYP82A24 were assayed using recombinant yeast microsomes. Both CYP71D106 and CYP71D145 demonstrated GS1 activity after prolonged incubation (5 h) with the microsomes and 4-dimethylallylglycinol; however, the specific activity of both P450s was only 1.7–2.1% of that of CYP71D8 (Supplementary Figure S5). CYP71D145 produced only glyceollin I in the enzyme assay, whereas CYP71D106 yielded glyceollin I and two other unknown compounds (P1 and P2). CYP82A24 also exhibited GS3 activity with 2-dimethylallylglycinol, producing unknown products (P3, P4, and P5); however, its GS3 activity was very low (0.5%) compared with that of CYP82A2. The retention times of P1 to P5 did not coincide with those of glyceollins I, II, or III.

### Expression analysis of *CYP71D8* and *CYP82A2*

**Figure 4:**
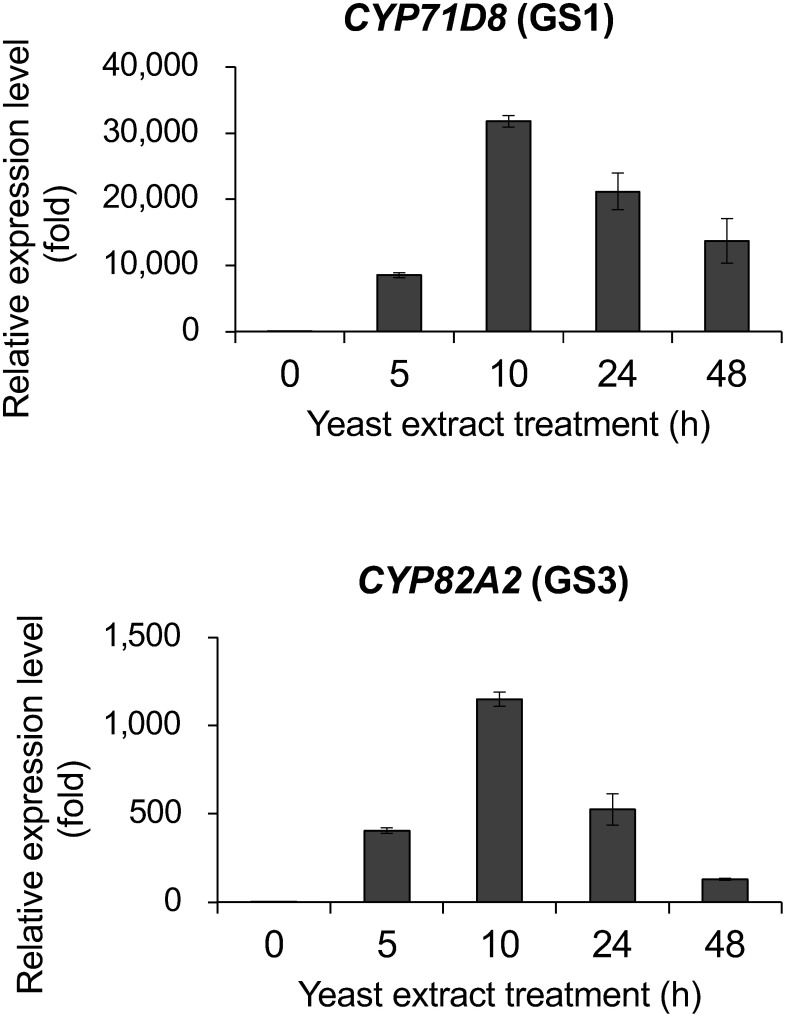
Figure 4. Expression analysis using real-time PCR. Data are expressed as mean±SE (*n*=3 biological replicates). Transcript levels were analyzed using the ΔΔCt method. *Skip16* was used as the internal standard, and transcript levels were normalized against the 0 h value, which was set at 1.

Phytoalexins are produced in response to biotic and abiotic stresses, and their biosynthetic genes are upregulated prior to the accumulation of these compounds (Uchida et al. 2017; Yoneyama et al. 2016). Comparing the time course of phytoalexin accumulation and transcript levels is important for understanding the biosynthesis. Previous studies have demonstrated that the accumulation of glyceollin I and III was induced by 0.3% (w/v) yeast extract in cultured soybean cells, with maximum content observed from 10 to 48 h after treatment (Akashi et al. 2009). The biosynthetic genes encoding *IFS*, *2-hydroxyisoflavanone dehydratase* (*HID*), *PTS*, *pterocarpan 6a-hydroxylase* (*P6aH*), *G4DT*, and *G2DT* were also transiently upregulated by elicitation (Uchida et al. 2017; Yoneyama et al. 2016). To confirm the involvement of both *CYP71D8* and *CYP82A2* in glyceollin biosynthesis, gene expression was analyzed using real-time PCR. Transient upregulation of both *CYP71D8* and *CYP82A2* transcripts was observed in the elicited soybean cells prior to glyceollin I and III accumulation (Figure 4). Furthermore, their expression was induced by fungal inoculation in soybean seedling in a previous study (Uchida et al. 2020).

## Discussion

CYP71D8 and CYP82A2 were initially identified as elicitor-inducible P450 genes in glyceollin-producing soybean cells (Schopfer and Ebel 1998). Both P450s exhibit organ-specific and distinct stress-induced expression patterns; however, the catalytic functions of the gene products have long been unidentified (Khatri et al. 2022; Xia et al. 2023; Zhu et al. 2009). Gene database analysis revealed that both *CYP71D8* and *CYP82A2* were co-expressed with the known genes involved in glyceollin biosynthesis. Furthermore, transient expression of *CYP71D8* and *CYP82A2* transcripts was detected in yeast extract treated soybean cells, prior to glyceollin I, and III accumulations (Figure 4). These results suggest that the both P450s are involved in glyceollin I and III biosynthesis in planta. Recently, the identification of P450 cyclases (GS1 and GS3) has been reported, also supporting the involvement of CYP71D8 and CYP82A2 in glyceollin biosynthesis (Khatri et al. 2024). Transcripts of paralogous genes (CYP71D106, CYP71D145, and CYP82A24) were also induced in soybean seedling by fungal inoculation (Supplementary Figure S6) (Uchida et al. 2020), but their involvement in glyceollin biosynthesis are currently unknown due to low enzymatic activities.

Several P450 proteins belonging to different subfamilies are involved in soybean phytoalexin biosynthesis, including CYP71D9 (flavonoid 6-hydroxylase, F6H), members of the CYP93C subfamily (IFS), the CYP81E subfamily (I2′H), and CYP93A1 (P6aH). IFS catalyzes the unusual oxidative aryl migration of a flavanone to form an isoflavonoid skeleton (Mizutani and Sato 2011), whereas other P450s exhibit simple hydroxylation reactions. Both GS1 and GS3 catalyze the dehydrogenative cyclization of a prenyl moiety and the C-3 hydroxyl of the A-ring, forming pyran and furan rings, which is an atypical reaction for P450 enzymes. Ether ring formation from a prenyl side chain and a hydroxyl group was also found in furanocoumarin biosynthesis. Angular and linear furanocoumarins are biosynthesized from demethylsuberosin (6-dimethylallylumberiferone) via sequential P450 reactions, and an epoxy intermediate has been postulated in the reaction (Larbat et al. 2007; Villard et al. 2021). Similar epoxy intermediate is assumed to be involved in the biosynthesis of cyclic ether ring-attached isoflavones in *Lupinus* spp. (Tahara and Ibrahim 1995). However, in soybeans, cyclization via an epoxide is not assumed to be involved in glyceollin biosynthesis (Welle and Grisebach 1988). Although it is unclear whether the unknown products (P1 to P5) produced by CYP71D106 and CYP82A24 are intermediates (Supplementary Figure S5), a detailed structural analysis of these products may reveal the mechanism.

This study revealed that CYP71D8 and CYP82A2 correspond to GS1 and GS3, respectively, and that multiple P450 enzymes are involved in the final step of the biosynthesis of glyceollin isomers. The identification of GS1 and GS3 in this study leaves glyceollin II synthase (GS2) as the only unidentified gene involved in major glyceollin biosynthesis (Figure 1). Cultured soybean cells treated with yeast extract did not produce glyceollin II, but treatment of soybean leaves with 1 mM copper(II) chloride increased accumulation of the compound and expression of *G2DT* (Yoneyama et al. 2016). The regulation of gene expression in the glyceolin II pathway is assumed to be different from that in the glyceollin I and III pathways. Cyclic ether ring-attached isoflavonoid phytoalexins are also found in bean (phaseollin), licorice (grabridin), *Pueraria* (tuberosin), and many other leguminous plants (Dewick 1986). Future studies identifying of GS2 in soybean and P450 cyclases in other plants are expected to elucidate the mechanism of formation of five- or six-membered cyclic ether rings.

## Abbreviations

CPR: cytochrome P450 reductase
F6H: flavonoid 6-hydroxylase
G2DT: (−)-glycinol 2-dimethylallyltransferase
G4DT: (−)-glycinol 4-dimethylallyltransferase
GS1: glyceollin I synthase
GS2: glyceollin II synthase
GS3: glyceollin III synthase
HID: 2-hydroxyisoflavanone dehydratase
I2′H: isoflavone 2′-hydroxylase
IFS: isoflavonoid synthase
P450: cytochrome P450
P6aH: pterocarpan 6a-hydroxylase
PTS: pterocarpan synthase

## Description of Supplementary Files

Supplementary Table S1. Primer sets using this study.

Supplementary Data S1. ^1^H-NMR data of CYP71D8 and CYP82A2 reaction products.

Supplementary Figure S1. HPLC chromatogram of the enzymatic reaction mixture of candidate P450s.

Supplementary Figure S2. Substrate specificities of CYP71D8 and CYP82A2.

Supplementary Figure S3. Michaelis–Menten plots.

Supplementary Figure S4. Alignment of amino acid sequences of soybean CYP71D and CYP82A subfamilies.

Supplementary Figure S5. HPLC chromatogram of the enzymatic reaction mixture of soybean P450s.

Supplementary Figure S6. The expression levels of P450 transcripts in fungal-inoculated soybean seedlings.
