# Supplementary material for: CYP71D8 and CYP82A2 catalyze the last committed step in biosynthesis of glyceollin isomers in soybean
Source: Plant Biotechnol (Tokyo). 2025 Mar 25;42(1):51–6. doi: 10.5511/plantbiotechnology.24.1113a (PMC12622905; doi:10.5511/plantbiotechnology.24.1113a)
Supplement: Supplementary Data [file plantbiotechnology-42-1-24.1113a-s001.pdf]

Supplementary Table S1. Primer sets using this study.

CYP71D8
Forward: AAAATGGAATATTCTCCATTGTCCATTG
Reverse: TCATGAAGCTTCATAAACAGTGGG

CYP71D145
Forward: ATAAAGCCATGGAACATTCTCAAC
Reverse: ATTAAAAAATATAAGTATCATGTAGCTTGG

CYP81E24
Forward: ATGGAAGGCAACCTTATCAAC
Reverse: TTAGAAAATCTTGCTAATGATCG

CYP82A2
Forward: AAAATGGAGTTAGTTCTAAACAGCAC
Reverse: TTAGATACTTTCATAACAACTAGGAG

CYP82A3
Forward: AAAATGGACCTTCTCCTAAATTGCC
Reverse: TTATAAAGTTTCATAATAGTTGGGAGAC

CYP82A4
Forward: AAAATGGAATTAGTTCTACATTTCCTAAAC
Reverse: TCACATACTTTTGTAACAACTTGGAG

CYP93A2
Forward: TGCACTCCATAGTTGAAGGTG
Reverse: TCACATAACAGGGAATGGGTTAAGC

CYP93A3
Forward: ATGGCTTTTCAAGTGTTGTTC
Reverse: TCATTTGGAGCGTCAATCACAC

CYP736A33
Forward: AAAATGTTACCTCAAACATTAGCCATCC
Reverse: TTAAGCTTTGATAAATAGGCGATAAGTTG

Realtime PCR

CYP71D8
Forward: GAAACCTGAGGATTTGGATATGG
Reverse: TCCCCAAAGTGTGGAGGTG

CYP82A2
Forward: TTCCATCACGGAGCTCTTCC
Reverse: CCGCAGAATCATGTTGAAGACA

Skip16
Forward: AAAAAGGTGCTGGGATAGAATAAAAA
Reverse: GGCCTCCGCTTCAGGAA

Supplementary Data S1. ^{1}H-NMR data of CYP71D8 and CYP82A2 reaction products.

For NMR identification of the reaction product, 0.5 mg substrates, 20 mg NADPH, and 5 mg recombinant yeast microsomes expressing CYP71D8 or CYP82A2 were used for the assay. After 7 h incubation, an ethyl acetate extract of the products was subjected to silica gel TLC (Kieselgel F254, Merck, Darmstadt, Germany) with hexane : ethyl acetate (1 : 1, v/v) as the solvent, and the reaction product was obtained.

CYP71D8 reaction product (Glyceollin I)
Substrate, 4-dimethylallylglycinol

^{1}H-NMR [(acetone-*d6*); δ: 1.35 (3H, *s*, H-4'), 1.39 (3H, *s*, H-5'), 4.11 (1H, *d*, *J* = 11.5 Hz, H-6), 4.19 (1H, *d*, *J* = 11.5 Hz, H-6), 5.27 (1H, *s*, H-11a), 5.66 (1H, *d*, *J* = 10.5 Hz, H-2'), 6.25 (1H, *d*, *J* = 2.5 Hz, H-10), 6.43 (1H, *dd*, *J* = 2.5, 8.0 Hz, H-8), 6.47 (1H, *d*, *J* = 8.0 Hz, H-2), 6.57 (1H, *d*, *J* = 10.5 Hz, H-1'), 7.22 (1H, *d*, *J* = 8.0 Hz, H-7), 7.24 (1H, *d*, *J* = 8.0 Hz, H-1).

CYP82A2 reaction product (Glyceollin III)
Substrate, 2-dimethylallylglycinol

^{1}H-NMR [(acetone-*d6*); δ: 1.73 (3H, *s*, H-5'), 3.00 (1H, *m*, H-1'), 3.37 (1H, *dd*, *J* = 9.7, 15.4 Hz, H-1'), 3.96 (1H, *d*, *J* = 11.5 Hz, H-6), 4.02 (1H, *d*, *J* = 11.5 Hz, H-6), 4.88 (1H, *m*, H-4'), 5.05 (1H, *m*, H-4'), 5.23 (1H, m, H-2'), 5.23 (1H, *s*, H-11a), 6.25 (1H, *d*, *J* = 2.5 Hz, H-10), 6.35 (1H, *s*, H-4), 6.42 (1H, *dd*, *J* = 2.5, 8.0 Hz, H-8), 7.16 (1H, *s*, H-1), 7.19 (1H, *d*, *J* = 8.0 Hz, H-7).

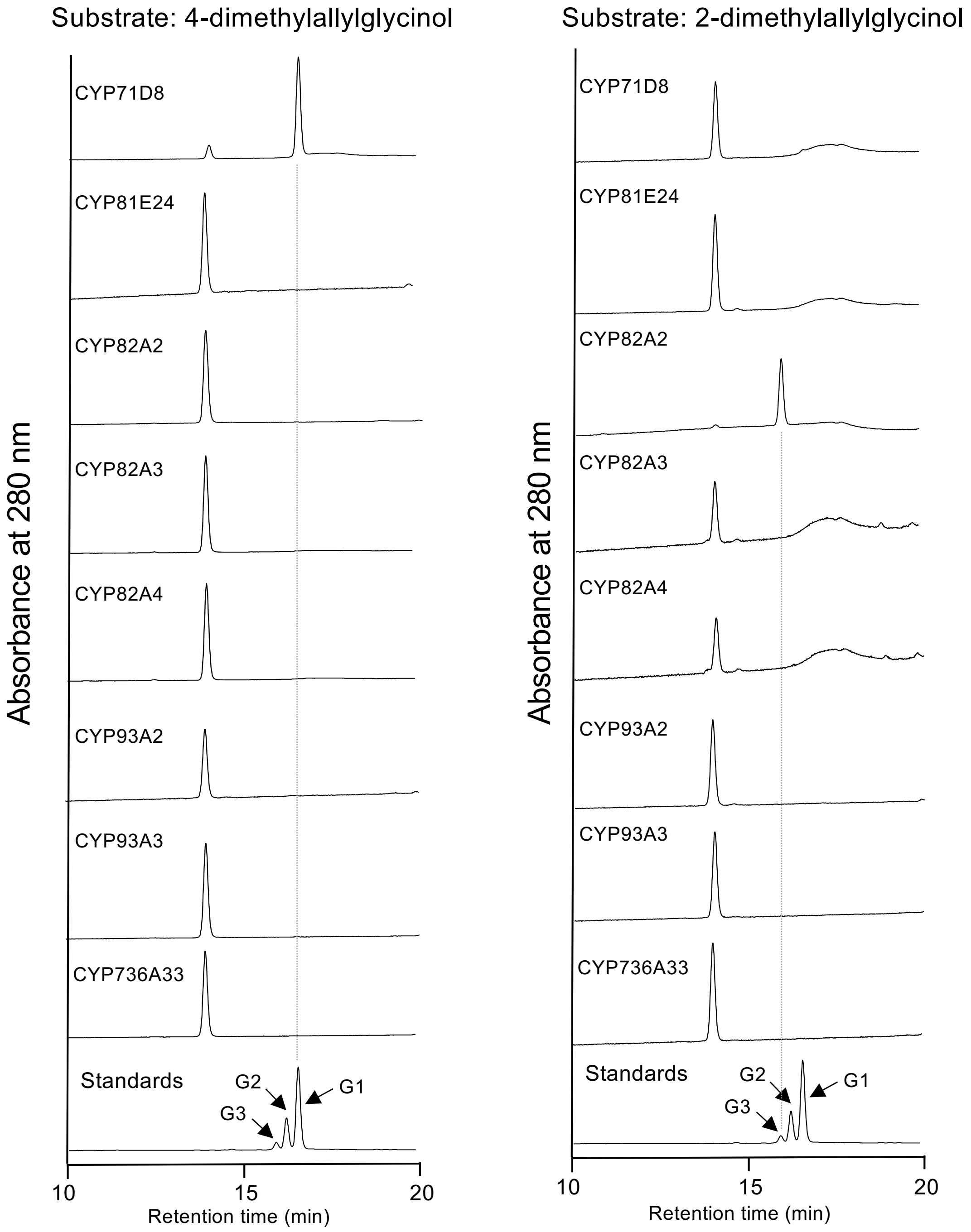

Supplementary Figure S1. HPLC chromatogram of the enzymatic reaction mixture of candidate P450s. Crude extracts (10,000 g supernatant) expressing P450s were reacted with a substrate (2- or 4-dimethylallylglycinol) in the presence of NADPH at 30°C for 24 h. The ethyl acetate extract of the reaction mixture was analyzed by HPLC. TSK-Gel ODS-80TM column was used for the analysis. Abbreviations used are: G1, glyceollin I; G2, glyceollin II; G3, glyceollin III.

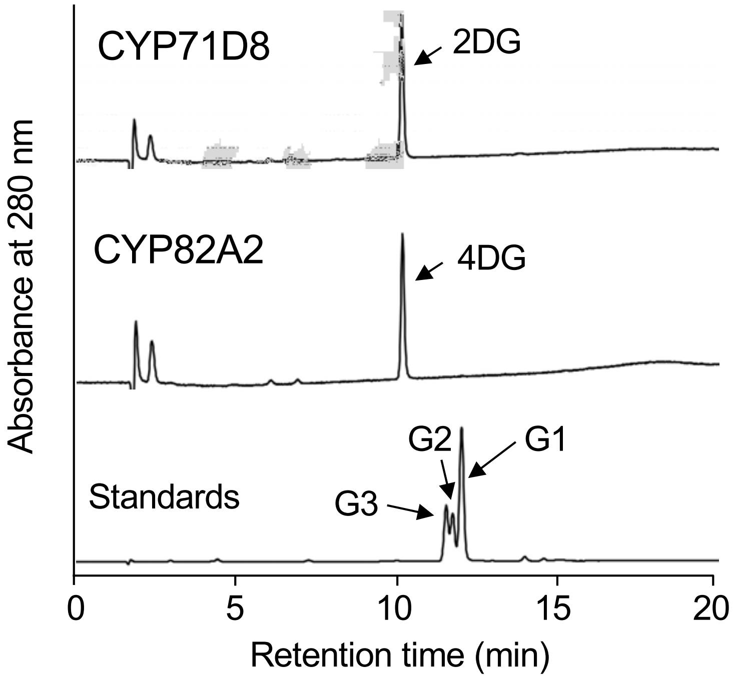

Supplementary Figure S2. Substrate specificities of CYP71D8 and CYP82A2. The yeast microsomes expressing P450s were reacted with a substrate (2- or 4-dimethylallylglycinol, 10 μg) in the presence of 1 mM NADPH at 30°C for 5 h. The ethyl acetate extract of the reaction mixture was analyzed by HPLC. The ordinate scales of the HPLC charts are equal. XBridge C18 column was used for the analysis. Abbreviations used are: 2DG, 2-dimethylallylglycinol; 4DG, 4-dimethylallylglycinol; G1, glyceollin I; G2, glyceollin II; G3, glyceollin III.

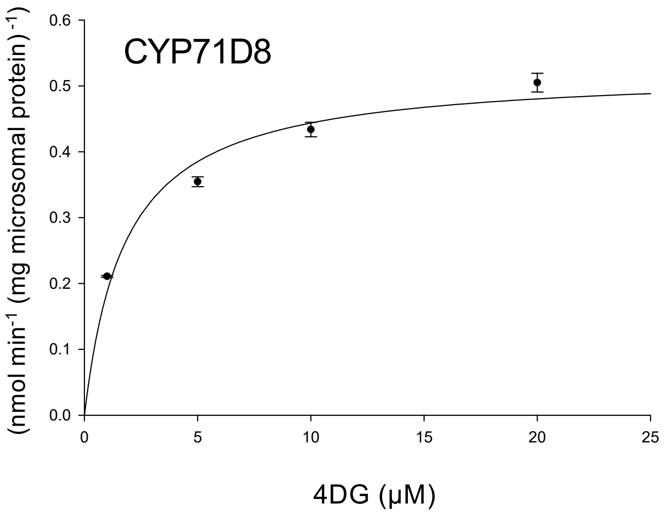

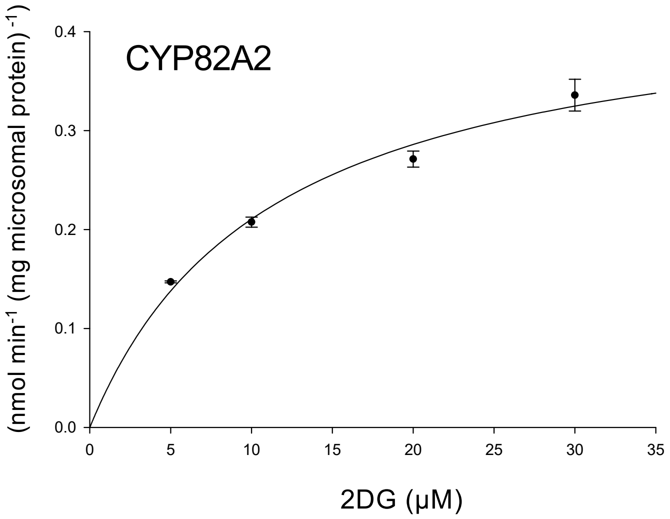

Supplementary Figure S3. Michaelis–Menten plots. Kinetic parameters were calculated from the plots with varying concentrations of varying concentrations (1 to 30 μM) of substrate and a fixed concentration of NADPH (2 mM). The values are three independent experiments.

A

```
CYP71D8      1 MEYSPLSIVITFFVFLLLHWLVKTYKQKSSHKLPPGPWRLPIIGNLHQLALAASLPDQALQKLVRKYGPLMHLQLGEIST
CYP71D106    1 .......................I.....RY....S...........................................A
CYP71D145    1 ..H.Q.............LR...NH.P...........K.........V.......HH.....A................

CYP71D8     81 LVVSSPKMAMEMMKTHDVHFVQRPQLLAPQFMVYGATDIAFAPYGDYWRQIRKICTLELLSAKRVQSFSHIRQDENKKLI
CYP71D106   81 ...........V...........................V....................................R...
CYP71D145   81 ...........I.....LA...........Y.A............E....M.........................R...

CYP71D8    161 QSIHSSAGSPIDLSGKLFSLLGTTVSRAAFGKENDDQDEFMSLVRKAITMTGGFEVDDMFPSLKPLHLLTRQKAKVEHVH
CYP71D106  161 .........S.............................L...............L........................
CYP71D145  161 ...Q..........S................NK..............VA......L..............G......EI.

CYP71D8    241 QRADKILEDILRKHMEKRTRVK-EGNGSEAEQEDLVDVLLRLKESGSLEVPMTMENIKAVIWNIFAAGTDTSASTLEWAM
CYP71D106  241 ...................IG.-..S-N......................................S....P........
CYP71D145  241 K...R.........V.....A.E...N...Q..........IQQ......Q..TGHV.....D.................

CYP71D8    320 SEMMKNPKVKEKAQAELRQIFKGKEIIRETDLEELSYLKSVIKETLRLHPPSQLIPRECIKSTNIDGYEIPIKTKVMINT
CYP71D106  319 .......R.R.........T................................................D...........
CYP71D145  321 A......R.R.....VI..A.....T...............................................S......

CYP71D8    400 WAIGRDPQYWSDADRFIPERFNDSSIDFKGNSFEYIPFGAGRRMCPGMTFGLASITLPLALLLYHFNWELPNKMKPEDLD
CYP71D106  399 .............E.......D.........................I............................A...
CYP71D145  401 .............E.......DG.Y.......Y...............................................

CYP71D8    480 MDEHFGMTVARKNKLFLIPTVYEAS
CYP71D106  479 ...L..L..V..........I....
CYP71D145  481 .N.......G.....C......Q.T
```

B

```
CYP82A2      1 MELVLNSTTIGVGVVSLILLLYLFLRGGSWKSGEEGPPTVAGAWPIIGHLPLLLGSKTPHKTLGDLADKYGPIFSIKIGA
CYP82A24     1 .D....T.............-....CRR.S...............................................L..

CYP82A2     81 KNAVVVSNWEMAKECYTTNDIAVSSLPDLISANLLCYNRSMIVVAPYGPYWRQLRKILMSEFLSPSRVEQLHHVRVSEVQ
CYP82A24    80 .....I.....................N..............L..........M..........................

CYP82A2    161 SSITELFRDWRSNKNVQSGFATVELKQWFSLLVFNMILRMVCGKRYFSASTSDDEKANRCVKAVDEFVRLAATFTVGDAI
CYP82A24   160 N...D..GA.......E..C.L...........................T.......K....................T.

CYP82A2    241 PYLRWFDFGGYENDMRETGKELDEIIGEWLDEHRQKRKMGENVQDLMSVLLSLLEGKTIEGMNVDIVIKSFVLTVIQAGT
CYP82A24   240 ............K.................E..............F.N..........................I...A.

CYP82A2    321 EASITTLIWATSLILNNPSVLEKLKAELDIQVGKERYICESDLSKLTYLQAVVKETLRLYPPAPLSRPREFEEDCTIGGY
CYP82A24   320 .......V......................................................G.................

CYP82A2    401 TVKKGTRLITNLSKIHTDHNVWSNPLEFKPERFLTTDKDIDMKGQHFQLLPFGGGRRICPGINLGLQTVRLTLASFLHSF
CYP82A24   400 .....................................................S.......V.......H..........

CYP82A2    481 EILNPSTEPLDMTEVFRATNTKATPLEILIKPRLSPSCYESI
CYP82A24   480 ................GV..S...S................M
```

Supplementary Figure S4. Alignment of amino acid sequences of soybean CYP71D and CYP82A subfamilies. Identical amino acid residues are represented in reverse type and shown as dots. Gaps (−) are inserted to optimize alignment. (A) Amino acid sequence of CYP71D8 aligned with CYP71D106 and CYP71D145. Identities at the amino acid level are as follows: CYP71D8 vs. CYP71D106 (94%), CYP71D8 vs. CYP71D145 (87%), CYP71D106 vs. CYP71D145 (84%). (B) Amino acid sequence of CYP82A2 aligned with CYP82A24. CYP82A2 is 92% identical to CYP82A24 at the amino acid level.

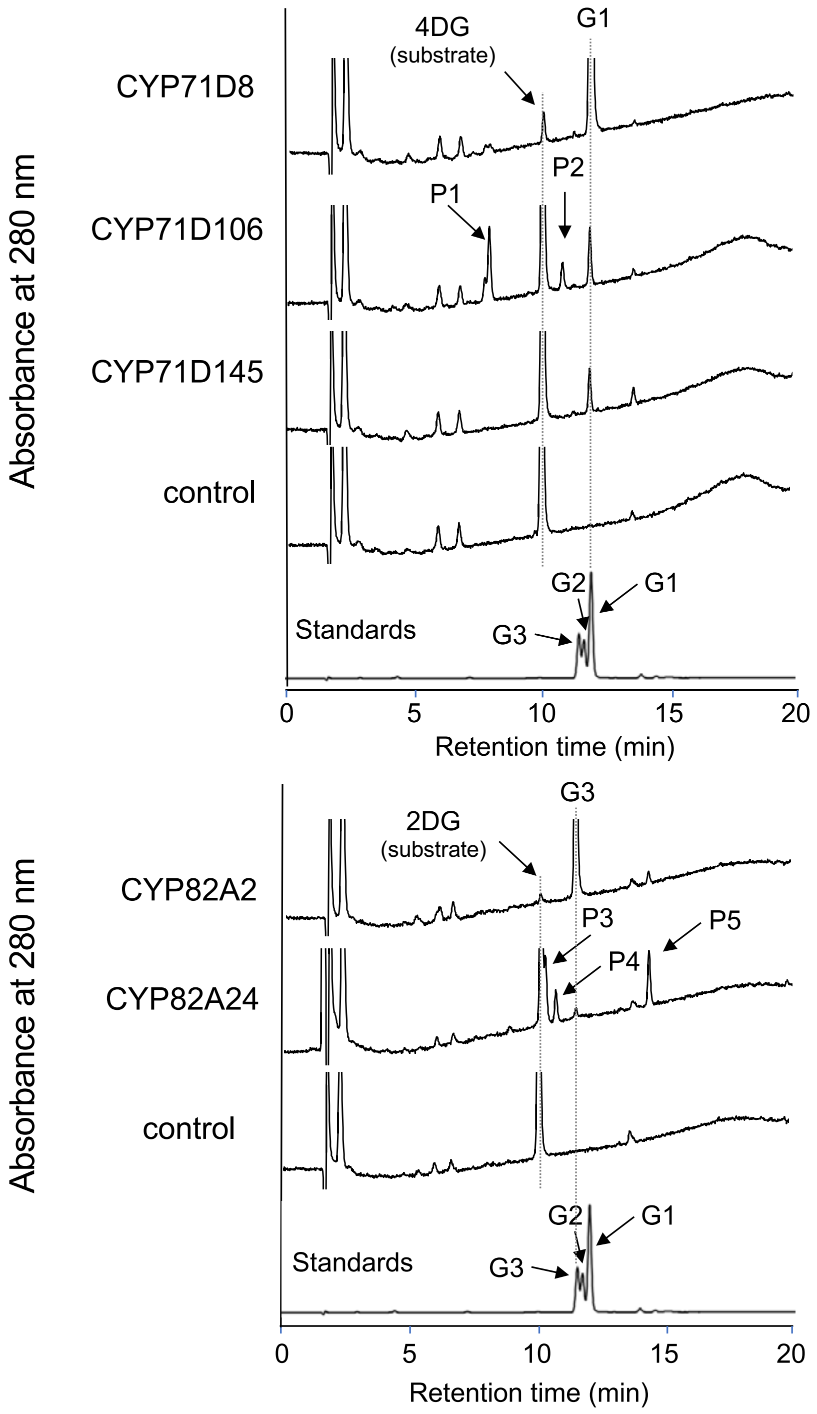

Supplementary Figure S5. HPLC chromatogram of the enzymatic reaction mixture of soybean P450s. The yeast microsomes expressing P450s were reacted with a substrate (2- or 4-dimethylallylglycinol) in the presence of NADPH for 5 h. The ethyl acetate extract of the reaction mixture was analyzed by HPLC. For the control, the microsomes of yeast cells transformed with pYES2 were used with the same substrates. The ordinate scales of the HPLC charts are equal. The eluates were monitored at 280 nm. Retention times are as follows: G1 (11.5 min), G2 (11.7 min), G3 (12.0 min), 2DG and 4DG (10.1 min), P1 (8.1 min), P2 (10.9 min), P3 (10.3 min), P4 (10.7 min), P5 (14.3 min). Abbreviations used are: 2DG, 2-dimethylallylglycinol; 4DG, 4-dimethylallylglycinol; G1, glyceollin I; G2, glyceollin II; G3, glyceollin III.

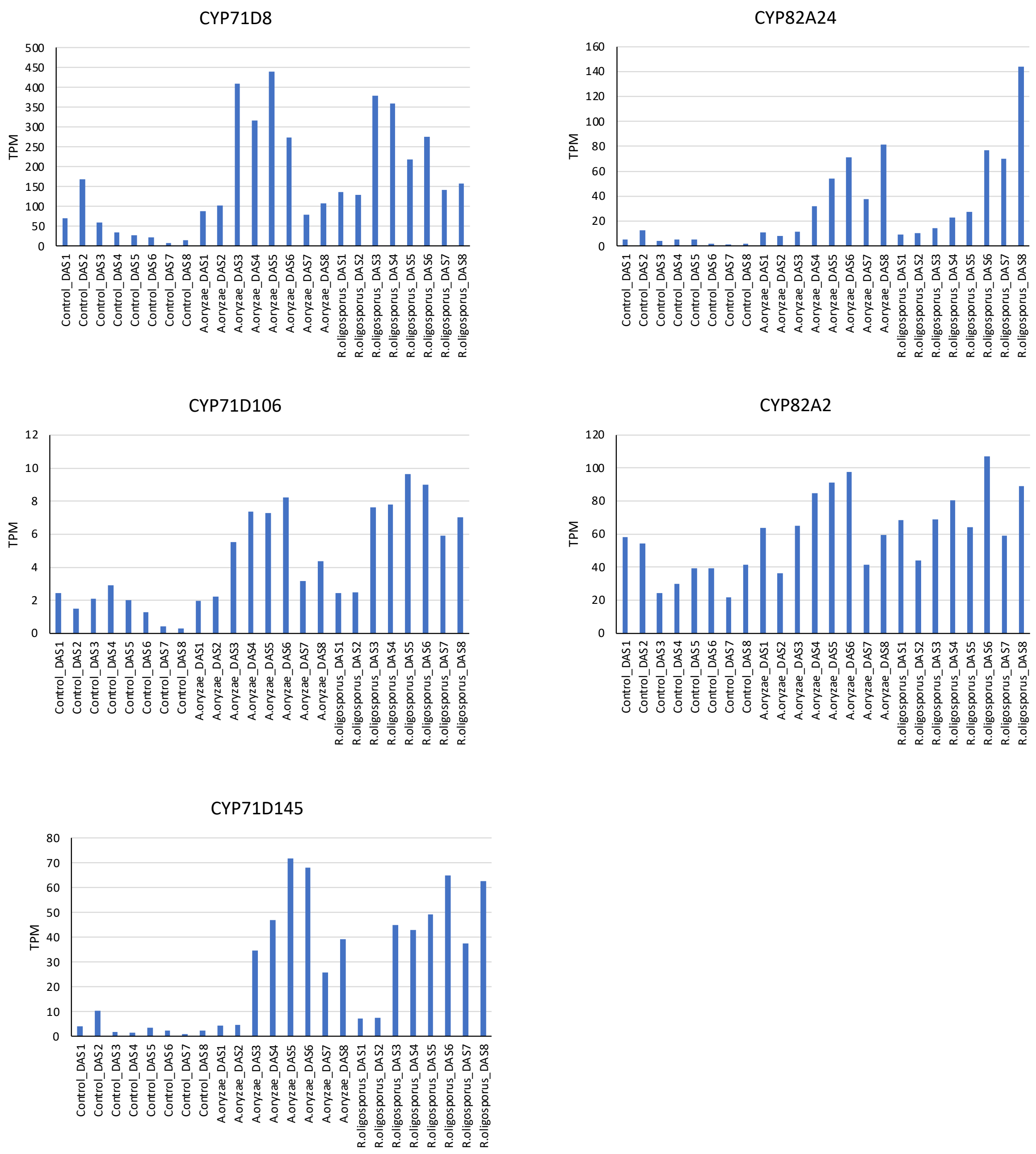

Supplementary Figure S6. The expression levels of P450 transcripts in fungal-inoculated soybean seedlings. TPM (transcripts per million) values were extracted from Supplementary Table S3 and S4 of an article (Uchida K, Sawada Y, Ochiai K, Sato M, Inaba J, Hirai MY (2020) Identification of a Unique Type of Isoflavone *O*-Methyltransferase, GmIOMT1, Based on Multi-Omics Analysis of Soybean under Biotic Stress. *Plant Cell Physiol* 61: 1974-1985). Two days old soybean seedlings were inoculated with spores of *Aspergillus oryzae* or *Rhizopus oligosporus.* DAS, days after soaking the seedlings in fungal spores.
